# The effects of negative emotions on sensory perception: fear but not anger decreases tactile sensitivity

**DOI:** 10.3389/fpsyg.2014.00942

**Published:** 2014-08-22

**Authors:** Nicholas J. Kelley, Brandon J. Schmeichel

**Affiliations:** Department of Psychology, Texas A&M University, College Station, TXUSA

**Keywords:** touch, emotion, sensory perception, tactile, fear

## Abstract

Emotions and sensory perceptions are closely intertwined. Of the five senses, sight has been by far the most extensively studied sense in emotion research. Relatively less is known about how emotions influence the other four senses. Touch is essential for nonverbal communication in both humans and other animals. The current investigation tested competing hypotheses about the effect of fear on tactile perception. One hypothesis based on evolutionary considerations predicts that fear enhances sensory perception, including tactile sensitivity. A competing hypothesis based on research on peripheral psychophysiology predicts that fear should decrease tactile sensitivity. Two experiments that induced negative emotional states and measured two-point discrimination ability at the fingertip found that fear reduces tactile sensitivity relative to anger or a neutral control condition (Studies 1 and 2). These findings did not appear to be driven by participants’ naïve beliefs about the influence of emotions on touch (Study 3). The results represent the first evidence of the causal impact of emotional states on tactile sensitivity, are consistent with prior evidence for the peripheral physiological effects of fear, and offer novel empirical grounds for developing and advancing theories of emotional influences on sensory perception.

## INTRODUCTION

When a person encounters a threat they may experience fear. Fear has been associated with heightened arousal, negative, or aversive subjective experience, and a recognizable facial expression including widened eyes and an open mouth. Research has indicated that fearful stimuli enhance visual perception. Specifically, fear-inducing stimuli (e.g., snakes) are located faster than fear-irrelevant stimuli in an array of distracter images (Öhman et al., 2001), and fear-inducing stimuli relative to neutral stimuli have been found to induce larger event-related potentials in the primary visual cortex a mere 90 ms after stimulus presentation (Stolarova et al., 2006). The enhancements in visual processing of frightening stimuli would appear to stem, at least in part, from the widening of the eyes associated with the characteristic facial expression of fear. The current investigation examined whether fear influences sensory perception in another channel—touch.

According to evolutionary perspectives on emotions, one of the functions of fear is to enhance perception (e.g., Susskind et al., 2008). Evidence lends support to this view insofar as the fear expression increases one’s visual field, speeds up eye movements, and increases nasal volume and air velocity during inspiration. Presumably this enhancement in perception facilitates more effective or efficient responding to fear-eliciting events. Does fear enhance perception in other sensory channels as well (in addition to the observed effects on vision and olfaction)? We tested the hypothesis that fear enhances sensory perception focusing specifically on the sensation of touch.

The opposite prediction could also be made. The hypothesis that fear reduces tactile sensitivity can be derived from evidence of the peripheral psychophysiological correlates of negative emotions. Specifically, research has observed that fear-related responses (e.g., responses to real or perceived threats) tend to be associated with increased vascular resistance, (e.g., Tomaka et al., 1993; Levenson, 2003; Hayashi et al., 2009) and decreased peripheral temperature, (Ekman et al., 1983; Collet et al., 1997). Vasodilation and decreased peripheral temperature have been observed to decrease tactile sensitivity (e.g., Provins and Morton, 1960). Hence, it may be the case that the peripheral physiological consequences of the emotion of fear produce decrements in tactile sensitivity.

## STUDY 1

As a first test of the effect of fear on tactile sensitivity, we experimentally induced different emotional states (fear, anger, or neutral) using an emotional memory task and then measured tactile sensitivity. We included anger as a comparison emotion for two reasons. First, research on peripheral psychophysiology has distinguished between the effects of fear, which causes vasoconstriction, and the effects of anger, which causes vasodilation. For instance, anger has been found to increase finger temperature whereas fear has been shown to decrease finger temperature (e.g., Levenson et al., 1991).These differences may be crucial for tactile sensation. Second, like fear, anger is characterized by circumplex models of emotion as a high arousal negative emotion (e.g., Russell, 1980). Thus, insofar as anger and fear have different effects on tactile sensitivity, we can discount the idea that the difference is due to arousing negative affect.

### METHOD

#### Participants and design

This experiment and the subsequent experiments were approved by the local institutional review board. Ninety-one undergraduate psychology students at Texas A&M University participated in Study 1 in exchange for credit toward a course requirement. Two participants were excluded for not following directions on the emotional memory task and one participant was excluded for having incomplete data, leaving 88 participants (55 female, 33 male) for analysis. Participants’ ages ranged from 18–23 (*M* = 19.05, SD = 1.09), and the ethnic composition of the sample was 69.3% Caucasian, 15.9% Latino/Hispanic, 8.0% Asian American, 3.4% African American, 1.1% American Indian/Alaskan Native, and 2.3% more than one race. These demographic factors did not influence the analyses reported below. Participants were randomly assigned among three emotional memory conditions: fear (*N* = 31), anger (*N* = 30), or neutral (*N* = 27). After retrieving the target emotional memories, tactile sensitivity was assessed using a two point discrimination task.

#### Procedure

Upon arrival to the laboratory participants learned that the purpose of the study was to examine the relationship between personality, emotions, and tactile sensitivity. After providing informed consent and completing demographic questionnaires participants completed a series of trait measures: the behavioral inhibition sensitivity and behavioral approach sensitivity scales (BIS/BAS; Carver and White, 1994), the three domain disgust sensitivity questionnaire (TDDS; Tybur et al., 2009), the UCLA loneliness scale (Russell et al., 1980), and the Southampton nostalgia scale (Routledge et al., 2008). These measures were included on an exploratory basis to examine the extent to which they may moderate the effect of negative emotions on tactile sensitivity^1^. Participants also completed a baseline measure of self-reported emotional states that included the following items: *afraid, tranquil, amused, active, scared, angry, glad, desire, alert, nervous, bored, content, depressed, hopeless, determined, pleasant, irritated, enthusiastic, hostile, pleased, mad, excited, guilty, calm, sad, attentive, down, happy, interested, ashamed, proud, strong, good mood, joyful, frustrated,* and *satisfied*. These items were rated on a scale from 1 = *very slightly/not at all* to 5 = *extremely*. Most relevant for present purposes are the anger items (*angry*, *frustrated*, *hostile, irritated,* and *mad*) and the fear items (*afraid* and *scared*).

Next, participants completed an autobiographical recall task (e.g., Forgas, 1999) designed to elicit one of three target emotions. In the *anger condition* participants were instructed to “Think of a time when you felt so angry that you wanted to explode (e.g., someone insulted you or took something that belonged to you).” In the *fear condition* participants were instructed to “Think of a time you were in danger or felt so afraid you wanted to run away (e.g., you came in contact with an animal you are afraid of or someone threatened you with physical violence).” In *the neutral condition* participants were instructed to “Think of a time when nothing out of the ordinary happened and you felt relatively neutral.” All participants were allotted 10 min to retrieve, relive, and write down their memories. Immediately following the memory task, participants completed the same self-report measure of emotional states as they did prior to the memory task.

**FIGURE 1:**
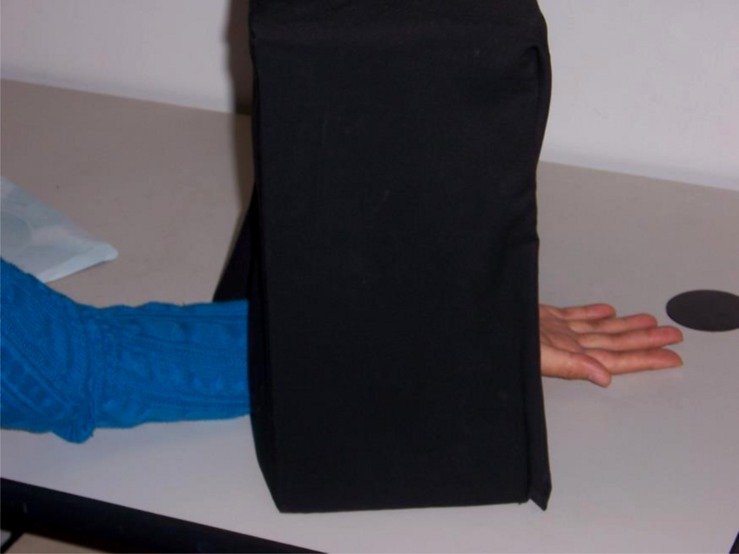
**Set-up for two-point discrimination task (Studies 1 and 2).** The black box prevented participants from seeing their hands.

Two-point discrimination refers to the ability to differentiate between one versus two points of sensation. The two-point discrimination task is implemented using a plastic pinwheel-shaped device with eight pairs of points ranging from 2 mm apart to 25 mm apart. Participants received 20 pokes (10 per index finger, counterbalanced) with a random selection of one point of sensation (*n* = 6 total; 3 per hand) or two points of sensation (*n* = 14 total; 7 per hand) varying between 2 mm and 8 mm apart. This distance distribution was selected because it reflects normal sensory ability in the fingers (Hunter et al., 1995; Dellon, 1997). After each of the 20 pokes, participants stated whether they had experienced one point of sensation or two. To ensure that participants relied on tactile perception alone, they inserted their hand through a cardboard chamber that kept the hand out of sight during the task (see **Figure 1**). More errors on the two-point discrimination task indicate poorer tactile sensitivity.

Participants then completed a brief self-report measure of pain sensitivity, which was included in an exploratory fashion to assess the possibility that the emotion inductions altered self-reported pain sensitivity (which could have influenced responding on the tactile sensitivity test). Specifically, participants considered how painful each of 17 hypothetical scenarios would be (e.g., “Imagine you trap your finger in a drawer”) using a scale from 0 = *no pain* to 10 = *the most severe pain that you can imagine or consider possible* (see Ruscheweyh et al., 2009). The 17 items were averaged to create a composite score of pain sensitivity (*M* = 3.63, SD = 1.46, α = 0.94).

### RESULTS

#### Manipulation checks

Prior to the emotional memory task, participants reported how afraid and scared they were. We combined responses to these two items (*r* = 0.87) into a single average score to represent fear. Baseline fear was low (*M* = 1.26, SD = 0.65) and did not differ across conditions, *F*(2,85) = 0.20, *p* = 0.82. The average rating of these two items assessed after the emotional memory task (*r* = 0.88) indicated that the emotional manipulation successfully influenced participants fear levels when controlling for baseline fear, *F*(2,85) = 4.10, *p* < 0.02, η^2^ = 0.09. *Post hoc* analyses revealed that participants were more afraid after writing about a fearful experience (*M* = 2.21, SD = 1.30) relative to writing about an anger-inducing (*M* = 1.52, SD = 0.71) or neutral experience (*M* = 1.46, SD = 0.83), *p*s < 0.008. The anger and neutral conditions did not differ, *p* > 0.72.

Participants also reported how angry, irritated, mad, frustrated, and hostile they felt before (α = 0.86) and after (α = 0.94) the emotional memory task. Baseline anger was low (*M* = 1.42, SD = 0.67) and did not differ across conditions, *F*(2,85) = 1.60, *p* = 0.21. The average post-manipulation ratings of these five items, controlling for baseline anger, indicated that the emotional memory manipulation successfully influenced participants’ anger levels, *F*(2,85) = 11.40, *p* < 0.001 η^2^ = 0.21. *Post hoc* analyses revealed that participants were more angry after writing about an angry experience (*M* = 2.63, SD = 1.18) relative to writing about a fearful (*M* = 1.83, SD = 1.12) or neutral experience (*M* = 1.33, SD = 0.53), *p*s < 0.004. Post-manipulation anger, when controlling for pre-manipulation anger, did not differ between the fear and neutral conditions, *p* = 0.10. Thus, the emotional memory task successfully elicited the target emotional states.

#### Tactile sensitivity

**FIGURE 2:**
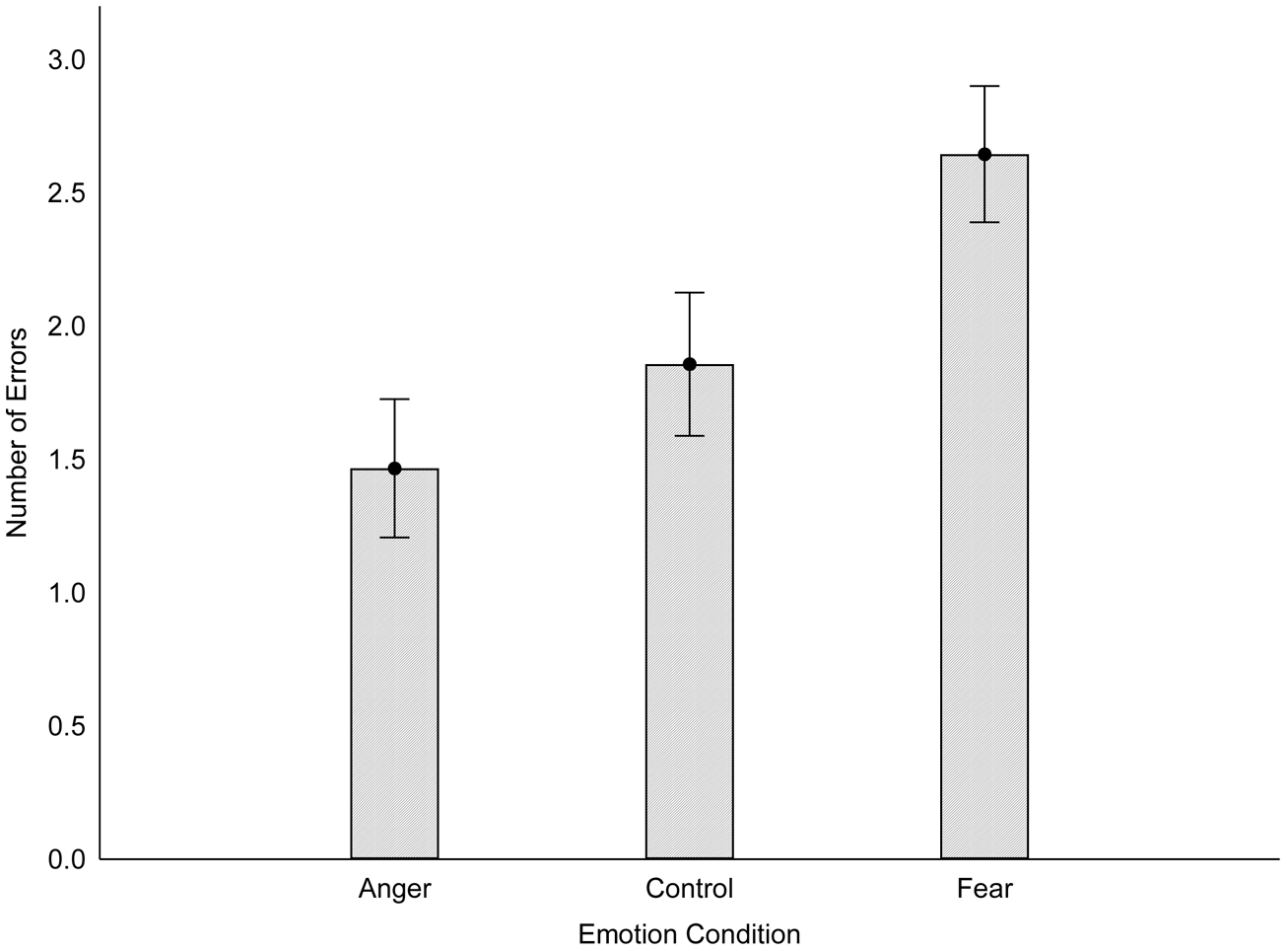
**Performance on the two-point discrimination task as a function of emotion condition (Study 1).** Higher scores indicate poorer tactile sensitivity. Error bars reflect standard errors.

A one-way analysis of variance (ANOVA) revealed that the type of emotional memory participants recalled significantly affected their two-point discrimination ability, *F*(2,85) = 5.34, *p* = 0.007, η^2^ = 0.11. *Post hoc* analyses revealed that participants in the fear condition made significantly more errors (*M* = 2.65, SD = 1.72) on the two point discrimination task (i.e., less tactile sensitivity) relative to participants who recalled an angry (*M* = 1.47, SD = 1.28) or neutral (*M* = 1.86, SD = 1.78) memory, *p*s = 0.002 and 0.047, respectively. The angry and neutral conditions did not differ, *p* = 0.54. The results are displayed in **Figure 2**.

Note that participants could make two types of errors on the two point discrimination task. Specifically, participants could mistake one point of sensation for two, or they could mistake two points of sensation for one. Further, the likelihood of mistaking two points of sensation for one was much higher than the likelihood of mistaking one point of sensation for two, because many more trials featured two points of sensation (14 trials) versus one point of sensation (6 trials). Indeed, only five participants (5.6% of the sample) mistook one point of sensation for two on at least one trial, whereas 72 participants (82% of the sample) mistook two points of sensation for one on at least one trial.

#### Pain sensitivity

We also assessed the extent to which the emotional memory manipulation influenced participants self-reported pain sensitivity and whether differences in self-reported pain sensitivity related to tactile sensitivity. The emotion manipulation did not influence self-reported pain sensitivity, *F*(2,85) = 0.90, *p* = 0.41. Further, pain sensitivity was not associated with number of errors on the two point discrimination task, *r*(86) = 0.17, *p* = 0.13.

### DISCUSSION

Study 1 found that fear reduces tactile sensitivity. Specifically, participants induced to experience fear exhibited reduced tactile sensitivity relative to participants who recalled angering or neutral memories. This pattern is consistent with prior evidence of the effects of fear on the peripheral nervous system, including decreased finger temperature (e.g., Levenson et al., 1991) and increased vascular resistance (e.g., Hayashi et al., 2009), which have been associated with reduced tactile sensitivity (e.g., Provins and Morton, 1960). The findings from Study 1 do not support the view that fear enhances sensory perception generally, although enhancements in visual perception and olfaction have been observed in previous research. We conducted a second study using a different method of emotion induction to try to replicate the effect of fear on tactile perception.

## STUDY 2

This study was a conceptual replication of Study 1 using pictures instead of memories to induce emotional states. Using a different method of emotion induction in Study 2 allowed us to assess the generalizability of the effect of fear on tactile sensitivity and ensure that the effect is not specific to idiosyncratic features of the emotional memory task. Based on the results from Study 1, we predicted that viewing fear-inducing pictures would reduce tactile sensitivity relative to viewing anger-inducing pictures or neutral pictures.

### METHOD

#### Participants and Design

One-hundred thirty-six undergraduate psychology students at Texas A&M University participated in exchange for credit toward a course requirement. Demographic information was not gathered from this sample. Fourteen participants were excluded from analyses because the computer shutdown due to overheating during the image viewing task, leaving 122 participants for analysis. Participants viewed images selected to induce anger (*N* = 41), fear (*N* = 38), or a neutral mood (*N* = 43) in a between-subjects experimental design. The pictures were borrowed from the International Affective Picture System (IAPS; Lang et al., 2008) and the Internet. After viewing a block of 32 images, tactile sensitivity was assessed using the same two-point discrimination task as in Study 1.

#### Procedure

As in Study 1, participants were told that the purpose of the study was to examine the relationship between personality, emotions, and tactile sensitivity. After providing informed consent participants completed the same personality questionnaires as in Study 1. Participants also completed a baseline measure of self-reported emotional states that included the following items: *afraid, tranquil, amused, active, scared, angry, glad, desire, alert, nervous, bored, content, depressed, hopeless, determined, pleasant, irritated, enthusiastic, hostile, pleased, mad, excited, guilty, calm, sad, attentive, down, happy, interested, ashamed, proud, strong, good mood, joyful, frustrated,* and *satisfied*. These items were rated on a scale from 1 = *very slightly/not at all* to 5 = *extremely*. The anger items included *angry*, *frustrated*, *hostile*, *irritated* and *mad*. The fear items included *afraid* and *scared*.

Next, participants viewed a series of images selected to elicit fear, anger, or a neutral mood state, respectively. The procedure for the image-viewing task and the anger condition pictures were adopted from Gable and Poole (2014). Pictures in the *anger condition* depicted anti-patriotic scenes (e.g., flag-burning; borrowed from Gable and Poole, 2014). Pictures in the *fear condition* depicted scenes of threat (e.g., snakes) and were borrowed from the IAPS [IAPS Picture Numbers: 1050, 1052, 1120, 1300, 1525, 1930, 1932, 2120, 2692, 2811, 3500, 5970, 5971, 6190, 6243, 6250, 6260, 6350, 6510, 6550, 6562, 6570, 6571, 6940, 8480, 8485, 9230, 9426, 9600, 9622, 9630, and 9670]. Pictures in the *neutral condition* depicted people or objects and were matched to the presence of objects (e.g., buildings) and faces in the fear and anger conditions. Neutral images were taken from the IAPS [IAPS Picture Numbers: 2190, 2210, 2215, 2396, 2440, 2441, 2493, 2499, 2516, and 2595] and the Internet^2^. Participants viewed 32 images of one of the picture types. Picture sizes (1024 × 768) were equivalent across conditions. All pictures were presented in the center of a 20-inch computer monitor with a gray background. Each trial consisted of a fixation cross (1000 ms) followed by a picture (6000 ms).

Immediately following the image-viewing task participants completed the same two-point discrimination task as in Study 2. After the two-point discrimination task participants re-viewed the slideshow and rated their emotional state using items related to the specific emotions of interest plus other filler items borrowed from the Positive and Negative Affect Schedule (PANAS-X; Watson and Clark, 1994). Specifically, participants rated on a scale from 1 = *very slightly/not at all* to 5 = *extremely* the extent to which the slideshow made them feel *afraid*, *angry*, *alert*, *bored*, *content*, *pleasant*, *mad*, *calm*, *sad*, *happy*, *irritated*, and *satisfied*. Most relevant for the present study are the fear item (*afraid*) and the anger items (*angry*, *mad*, and *irritated*).

### RESULTS

#### Manipulation checks

Baseline fear was low (*M* = 1.30, SD = 0.64) and did not vary by condition, *F*(2,119) = 0.35, *p* > 0.70. Post-manipulation fear was assessed with the fear-relevant item (*afraid*) on the post-manipulation state emotion scale. The picture-viewing manipulation successfully influenced participants post-manipulation self-reported fear, *F*(2,119) = 94.47, *p* < 0.001 η^2^ = 0.62. *Post hoc* analyses revealed that participants reported more fear after viewing pictures in the fear condition (*M* = 3.76, SD = 1.10) relative to the anger condition (*M* = 2.63, SD = 1.02) or neutral condition (*M* = 1.09, SD = 0.37), *p*s < 0.001. The anger condition also induced more fear than the neutral condition, *p* < 0.001.

Baseline anger was low (*M* = 1.39, *p* = 0.48) and did not vary as a function of the emotion manipulation, *F*(2, 119) = 0.83, *p* = 0.44. Post-manipulation anger was a composite of participants’ responses to the items *angry*, *mad*, and *irritated* (α = 0.94). The picture-viewing manipulation successfully influenced participants post-manipulation anger when controlling for baseline anger, *F*(2,119) = 122.61, *p* < 0.001 η^2^ = 0.68. *Post hoc* analyses revealed that participants were more angry after viewing pictures in the anger condition (*M* = 4.11, SD = 0.76) relative to the fear condition (*M* = 2.79, SD = 1.23) or neutral condition (*M* = 1.14, SD = 0.51), *p*s < 0.001. The fear condition also induced more anger than the neutral condition, *p* < 0.001. These results indicate that the picture slideshows successfully elicited in the intended emotional states.

#### Tactile sensitivity

**FIGURE 3:**
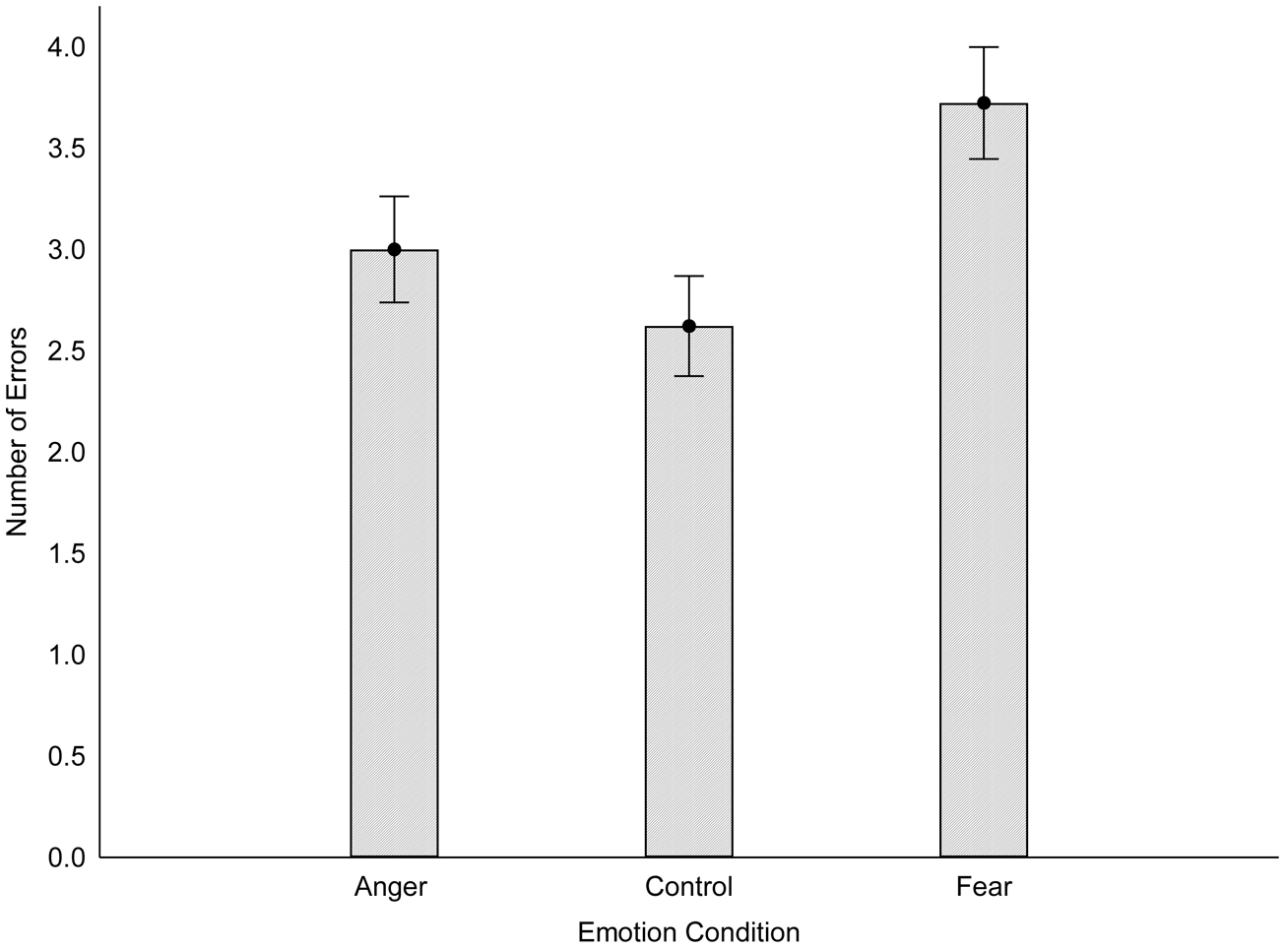
**Performance on the two-point discrimination task as a function of emotion condition (Study 2).** Higher scores indicate poorer tactile sensitivity. Error bars reflect standard errors.

Consistent with the findings from Study 1, a one-way ANOVA revealed that emotional picture type affected two-point discrimination, *F*(2,119) = 4.55, *p* = 0.01, η^2^ = 0.07. *Post hoc* analyses revealed that participants made significantly more errors (i.e., less tactile sensitivity) after viewing fear-inducing pictures (*M* = 3.71, SD = 1.69) relative to viewing anger-inducing (*M* = 2.85, SD = 1.68) or neutral pictures (*M* = 2.65, SD = 1.60), *p*s = 0.023 and 0.005, respectively. The anger and neutral groups did not differ, *p* = 0.58. Please see **Figure 3**.

As in Study 1, the majority of trials included two points of sensation, making the error of perceiving two points of sensation as one much more likely than mistaking one point of sensation as two. Indeed, only five participants (3.7% of the sample) mistook one point of sensation for two on at least one trial in Study 2, whereas 126 participants (92.6% of the sample) mistook two points of sensation for one on at least one trial.

### DISCUSSION

Study 2 replicated the effect observed in Study 1 using a different emotion induction. Compared to angry or neutral emotional states, viewing fear-inducing images reduced tactile sensitivity.

## STUDY 3

Studies 1 and 2 found that common laboratory-based methods of fear induction reduce tactile sensitivity relative to angry or neutral emotional inductions. However, the results observed in the first two studies may reflect the influence of participants’ naïve beliefs rather than, or in addition to, any influence of emotional states. More specifically, participants in both studies were explicitly aware that the purpose of the experiment was to examine how emotions influence tactile sensitivity. Thus, participants’ beliefs about how emotions influence tactile sensitivity may have influenced their responses as much or more than any actual effects of emotions on tactile sensitivity.

Previous research has found that lay beliefs, the beliefs persons hold about themselves and the world, influence a variety of social, emotional and motivational responses (see Molden and Dweck, 2006). If participants tend to believe that fear reduces tactile sensitivity, then the results of the first two studies may be attributable at least in part to participants’ beliefs (or their desire to behave in a manner consistent with the lay belief). To address the possibility that the effects found in Studies 1 and 2 were driven by participants’ beliefs, we conducted a study assessing participants’ naïve beliefs about the impact of different emotions on tactile sensitivity.

### METHOD

#### Participants and procedure

Forty-six undergraduate students at Texas A&M University participated in exchange for extra credit in a psychology course. Demographic information was not gathered from this sample. Participants indicated how they believed each of five emotions (anger, fear, disgust, sadness, and happiness) influence tactile sensitivity using a scale from 1 = *less sensitive* to 7 *= more sensitive*. Note that the phrase “tactile sensitivity” was not defined for participants, nor did any of them express confusion as to its meaning.

### RESULTS

**FIGURE 4:**
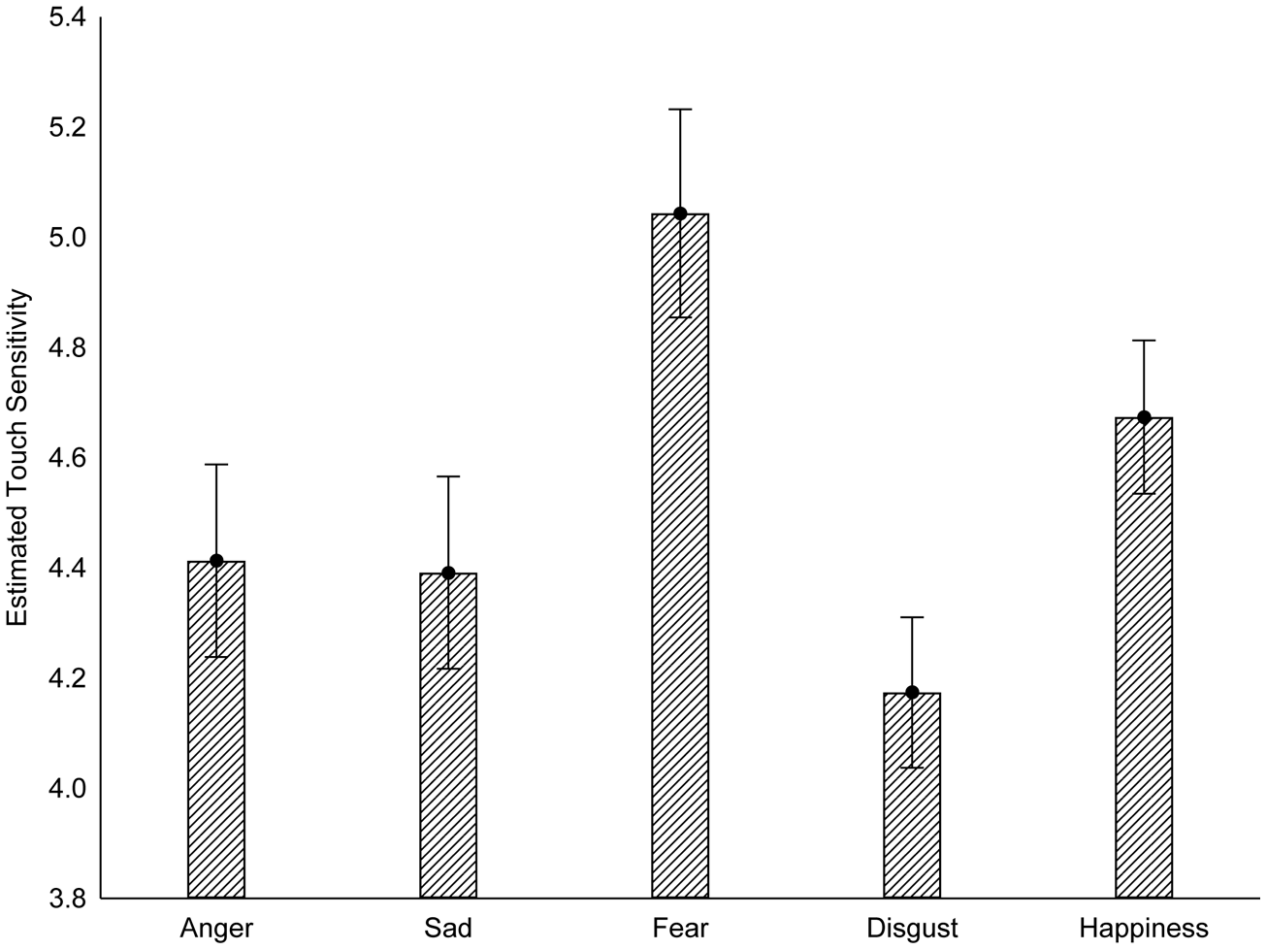
**Participants’ estimates of how different emotions would influence touch sensitivity.** Error bars reflect standard errors (Study 3).

Participants’ judgments of the effect of emotions on tactile sensitivity were analyzed using repeated-measures ANOVA. Results revealed a significant effect of emotion on perceived tactile sensitivity, *F*(4,180) = 4.49, *p* = 0.002, η^2^ = 0.09. *Post hoc* analyses revealed that participants believe fear increases tactile sensitivity relative to anger, disgust, and sadness (*p*s < 0.005), and marginally more than happiness (*p* = 0.098). Please refer to **Figure 4**.

### DISCUSSION

Study 3 identified lay beliefs about the effects of different emotions on tactile sensitivity. Participants believed that the experience of fear increases tactile sensitivity more than other negative emotions. These naïve beliefs run contrary to the results found in Studies 1 and 2, which suggests that those findings are not driven by participants’ naïve beliefs.

## GENERAL DISCUSSION

The current investigation examined the effects of fear on tactile sensitivity. We considered two opposing hypotheses. One hypothesis, consistent with evolutionary considerations (e.g., Susskind et al., 2008), was that fear increases tactile sensitivity. The second hypothesis, consistent with research on the peripheral psychophysiological correlates of fear, predicted the opposite effect, such that fear decreases tactile sensitivity. Two experiments found support consistent with the latter hypothesis. Using different methods of emotion induction, the two experiments found that fear decreases tactile sensitivity relative to angry or neutral states. Moreover, this effect did not appear to be driven by naïve beliefs about the relationship between fear and tactile perception: participants believed that fear would increase tactile sensitivity (Study 3), which is opposite to the patterns we observed in the first two experiments. The current investigation thus represents first evidence of the impact of fear on tactile sensitivity.

### IMPLICATIONS FOR EMOTION THEORY

The results suggest that different negative emotional states have different effects on tactile sensitivity. More specifically, fear has the effect of reducing tactile sensitivity but anger appears to have no effect. This pattern of findings has implications for dimensional theories of emotion. Two-dimensional models of emotion such as the circumplex model (e.g., Russell, 1980) conceptualize emotions as existing in a valence × arousal two-dimensional space. In such models, anger and fear are not differentiated (i.e., both are categorized as negatively valenced, high arousal emotions). The results of the current investigation do not accord well with a two-dimensional model of emotion. Rather, the current studies are more consistent with models of emotion that incorporate three or more dimensions, such as a three-dimensional model that view emotions as existing in a valence × arousal × motivational direction (approach vs. avoidance) three-dimensional space (e.g., Harmon-Jones and Allen, 1997, 1998). Models of emotion that incorporate motivational direction dissociate anger from fear insofar as anger is characterized by approach motivation whereas fear is associated with avoidance/withdrawal motivation. The current studies observed a distinction between fear and anger in the domain of touch perception and thus may be interpreted as consistent with a model of emotions that incorporates a dimension of motivational direction. Future research should examine the extent to which other avoidance-motivated emotions (e.g., disgust) also reduce tactile sensitivity.

Whereas most previous emotion research has focused on visual perception, the current experiments observed that the experience of emotion influences tactile perception. Evolutionary psychological theories of emotion (e.g., Cosmides and Tooby, 2000) suggest that emotions orchestrate the various processes of the mind and body into configurations suited to solve adaptive problems. One of these processes (among many) is sensory perception. From an evolutionary perspective, sensory perception is crucial for understanding the functions of emotion. Thus, by observing that emotions influence tactile perception, the current results offer additional empirical grounds for developing and advancing evolutionary psychological theories of emotion.

Although the current studies found evidence that fear reduces tactile sensitivity, which appears to contradict the evolutionary-inspired hypothesis that fear enhances perception (e.g., Susskind et al., 2008), the findings do not necessarily contradict the notion that fear has adaptive effects on perception. More simply, it may be the case that the experience of fear does not indiscriminately boost perception. Future research should refine evolutionary theories of the effects of emotions on perceptions to consider why reduced tactile sensitivity might be an adaptive aspect of fear response. One possibility is that reduced tactile sensitivity may be a consequence of the body’s tendency to preserve to preserve blood/energy in the central nervous system and the core of the body during fear-inducing situations.

Another possibility is that fear (and perhaps other emotional states) enhances some forms of sensory perception, but only at a cost in the sensitivity of other perceptual systems. The current evidence that fear reduces tactile sensitivity, when viewed in light of previous evidence that fear enhances visual perception (e.g., Schupp et al., 2004), is consistent with this view. Limited resource models of attention could be invoked to explain such a trade-off. Future research assessing the impact of different emotions on multiple sensory modalities is needed to test this hypothesis.

### TACTILE SENSITIVITY, TASK DIFFICULTY, AND TASK INTERFERENCE

One possible alternative explanation is that our results reflect the influence of negative emotions on making accurate judgments. Note, however, that we observed different effects for anger versus fear. Prior research suggests that anger makes decision makers less accurate, objective or rational (Bodenhausen et al., 1994; Lerner et al., 1998; Tiedens, 2001; Tiedens and Linton, 2001; Lerner and Tiedens, 2006; Small and Lerner, 2008). Angry individuals are more optimistic when making risk assessments whereas fearful individuals are more pessimistic (Lerner and Keltner, 2000, 2001). Insofar as a two-point discrimination task represents a decision-making task, research on angry decision-makers would lead us to expect anger to decrease performance on the two-point discrimination task. However, we found that two-point discrimination among participants in the anger condition did not differ from those in the control condition. Additionally, our results likely do not reflect a general decline in performance stemming from negative emotion; If that were the case, then we would have observed similar declines in both the fear and anger conditions, but we found decrements only in the fear condition.

It is also important to note the timing of the dependent measure in relation to the emotion manipulation. Participants completed the two-point discrimination task only after the emotion induction. One alternative approach would have been to conduct the two-point discrimination task concurrently with the emotion induction. Because fear-relevant stimuli capture attention and enhance visual processing (e.g., Öhman et al., 2001; Susskind et al., 2008), an effect of fear on reduced tactile stimuli in such a design could have been interpreted as evidence of task interference rather than a decrement in tactile perception. Likewise, measuring the dependent measure concurrent with the emotion induction likely would have dampened the effect of the emotion induction (because attention to the fear induction would have been distracted by performing the two-point discrimination task). By measuring tactile sensitivity immediately after the emotion induction, however, the findings in both Studies 1 and 2 are unlikely to be due to concurrent interference or distraction.

### ARE NAïVE BELIEFS ABOUT THE FEAR-TOUCH RELATIONSHIP MISGUIDED?

Fear and anger were induced with standard laboratory-based methods of emotion induction, including autobiographical recall (Study 1) and emotional picture viewing (Study 2), respectively. These methods reliably induced the target emotional states, but it seems likely that the anger and fear participants experienced in these studies were less arousing and impactful than the anger or fear individuals may experience in the course of their day-to-day lives. If that is correct, then perhaps the naïve beliefs participants expressed in Study 3 are not as misguided as the findings from Studies 1 and 2 seem to suggest. It may be that fear only decreases tactile sensitivity when the intensity of the fear is relatively mild (as may have been the case in Studies 1 and 2), whereas fear increases tactile sensitivity (consistent with the lay beliefs identified in Study 3) when the intensity of the fear is higher. Future research inducing more intense fear states is needed to test this hypothesis.

### IMPLICATIONS AND FUTURE DIRECTIONS

The current results are consistent with the idea that the relationship between fear and tactile sensitivity is mediated by changes in peripheral physiology. As noted previously, fear-related responses cause a decrease in peripheral temperature (Ekman et al., 1983; Collet et al., 1997), and decreases in peripheral temperature have been found to reduce tactile sensitivity (e.g., Provins and Morton, 1960). The current results taken together with prior work therefore suggest that changes in peripheral physiology may mediate the relationship between fear and decreased tactile sensitivity. Future research should address this possibility directly by assessing peripheral physiology in addition to tactile sensitivity.

Another fruitful avenue for further research may be to assess tactile perception with something other than two-point discrimination. In addition to the ability to distinguish one point of sensation from two, tactile sensations vary in other important respects (e.g., pressure, temperature, texture). Perhaps different emotions differentially impact sensitivity to different components of tactile sensations. Further, given the importance of touch in interpersonal communication (e.g., Hertenstein, 2002; Hertenstein et al., 2006a,b; Small and Lerner, 2008; Hertenstein and Keltner, 2011), more research is needed to understand the impact of emotions on sensitivity to and perception of human touch.

Lastly, the current research may have practical implications for the visually impaired community. Individuals who are visually impaired rely more on tactile perception to navigate the world around them compared to their sighted counterparts (e.g., Bhattacharjee et al., 2010). If fear decreases tactile sensitivity, then fear may interfere with navigational ability in the visually impaired. In the case of those visually impaired individuals who read Braille, tactile perception is essential for the acquisition of a great deal of information. Fear, by reducing tactile sensitivity, may make reading Braille more challenging and in turn may impair the acquisition and recall of information acquired through reading. Further research with visually impaired individuals is needed to trace potential practical implications of the current findings.

## CONCLUSION

A burgeoning literature attests to the impact of emotions on visual perception. Much less attention has been given to the effects of emotions on other sensory modalities. The current investigation focused on the sense of touch, which is central to development, communication, and many aspects of social life. The results revealed that although persons expect fear to increase tactile sensitivity, experimental inductions of fear states reduce tactile sensitivity. These findings pave the way for additional research on the impact of emotional states on sensory perception.

## Conflict of Interest Statement

The author declares that the research was conducted in the absence of any commercial or financial relationships that could be construed as a potential conflict of interest.

## Footnotes

1 The individual difference measures were included for exploratory purposes. Results pertaining to these measures are not reported here. Interested readers may contact the lead author for additional information.

2 Interested readers may contact the lead author for additional information regarding the Internet images used in Study 2.
